# 0391. Comparison of the histopathologic effects on the lungs of two external chest compression devices (lucas versus autopulse) in a swine model of ventricular fibrillation

**DOI:** 10.1186/2197-425X-2-S1-P24

**Published:** 2014-09-26

**Authors:** C Pantazopoulos, I Floros, A Mega, C Rigas, I Pavleas, P Vernikos, N Archontoulis, D Xanthis, N Iacovidou, T Xanthos

**Affiliations:** General Hospital of Athens Laiko, Intensive Care Unit, Athens, Greece; University of Athens, Medical School, Neonatology, Athens, Greece; University of Athens, Medical School, M.Sc. Programme in Cardiorespiratory Resuscitation, Athens, Greece

## Introduction

Given the difficulty of performing efficient CPR compressions, technology has turned to automaticity. LUCAS device has a pneumatically driven piston to compress the heart and uses active decompression suction on the upstroke. AUTOPULSE is a load distributing band compressor, that is mechanically actuated and battery driven. It provides both direct compression and semi-circumferential thoracic compression.

## Objectives

Lung injury may occur during cardiorespiratory resuscitation with external chest compression devices. Aim of this study is to compare 2 different external chest compression devices (LUCAS and AUTOPULSE) regarding differences in lung injury that they may cause.

## Methods

Forty (40) pigs were randomly allocated into 2 groups. Group L (LUCAS), n=20 and Group A (AUTOPULSE), n=20. After anesthesia, ventricular fibrillation was induced. Five minutes post-cardiac arrest without treatment, resuscitation was initiated. After resuscitation, lung biopsy via a mini-thoracotomy was obtained (right lung lower lobe).

## Results

Histopathology findings revealed a heterogeneous interstitial infiltrate and vascular congestion in all samples studied. There was no statistically significant difference between the two groups. (P>0.05)

## Conclusions

LUCAS and AUTOPULSE devices present no histopathological differences concerning lung injury after cardiorespiratory resuscitation.
